# De Novo transcriptome assembly and differential expression analysis of *catharanthus roseus* in response to salicylic acid

**DOI:** 10.1038/s41598-022-20314-4

**Published:** 2022-10-24

**Authors:** Narges Soltani, Farhad Nazarian Firouzabadi, Alireza Shafeinia, Masoud Shirali, Ayeh Sadat Sadr

**Affiliations:** 1 Production Engineering and Plant Genetics Department, Faculty of Agriculture and Natural Resources, Lorestan University, P.O. Box 465, Khorramabad, Iran; 2 Department of Plant Production & Genetics, Faculty of Agriculture, Agricultural Sciences & Natural Resources, University of Khuzestan, Mollasani, Iran; 3 Agri-Food and Biosciences Institute, Hillsborough, BT26 6DR UK; 4 School of Biological Sciences, Queen’s University Belfast, Belfast, BT9 5AJ UK; 5 South of Iran Aquaculture Research Institute (SIARI), Iranian Fisheries Science Research Institute, Agricultural Research Education and Extension Organization (AREEO), Ahvaz, Iran

**Keywords:** Biotechnology, Molecular biology

## Abstract

The anti-cancer vinblastine and vincristine alkaloids can only be naturally found in periwinkle (*Catharanthus roseus*). Both of these alkaloids' accumulations are known to be influenced by salicylic acid (SA). The transcriptome data to reveal the induction effect (s) of SA, however, seem restricted at this time. In this study, the de novo approach of transcriptome assembly was performed on the RNA-Sequencing (RNA-Seq) data in *C. roseus*. The outcome demonstrated that SA treatment boosted the expression of all the genes in the Terpenoid Indole Alkaloids (TIAs) pathway that produces the vinblastine and vincristine alkaloids. These outcomes supported the time-course measurements of vincristine alkaloid, the end product of the TIAs pathway, and demonstrated that SA spray had a positive impact on transcription and alkaloid synthesis. Additionally, the abundance of transcription factor families including bHLH, C3H, C2H2, MYB, MYB-related, AP2/ ERF, NAC, bZIP, and WRKY suggests a role for a variety of transcription families in response to the SA stimuli. Di-nucleotide and tri-nucleotide SSRs were the most prevalent SSR markers in microsatellite analyses, making up 39% and 34% of all SSR markers, respectively, out of the 77,192 total SSRs discovered.

## Introduction

*Catharanthus roseus,* a diploid (2n = 16) plant native to Madagascar, belongs to the Apocynaceae family. Despite the fact that it is grown as an ornamental plant in many tropical countries^1,2^, commercial alkaloids are rich in the aerial and underground tissues of this medicinal herb^3–5^.

A wide range of plant driven alkaloids such as ajmalicine, serpentine, vinblastine and vincristine have been extensively used in clinical therapy^1,6^. Regarding the plant kingdom, *C. roseus* solely synthesize vinblastine and vincristine alkaloids which are used in cancer treatments^7,8^. However, the synthesis rate and accumulation of both alkaloids in *C. roseus* are low and insufficient for the economic benefit of pharmaceutical industries. To this end, attempts to synthesize the aforementioned alkaloids, have not been successful yet^8–11^. The *C. roseus* valuable alkaloids are synthesized in the Terpenoid Indole Alkaloids (TIAs) pathway. It's interesting to note that the plant's developmental stages and the cell's reaction to biotic and abiotic stimuli alter the expression of TIAs pathway genes^7,8,12,13^. Many transcriptional activators and repressors regulate the transcription level of TIAs pathway genes^8,14–17^. Therefore, revealing the biosynthetic pathway of TIAs and identification of the regulators of these compounds can be useful in metabolic pathway engineering^1,8^. In recent years, several studies have been performed to identify genes and regulators of the TIAs pathway^1,7,8,15,16,18,19^. Despite tremendous advances, there is a need to understand and identify other genes and regulators involved in the TIAs pathway^8.^

The use of Next-Generation Sequencing (NGS) technology in analyzing genomic and transcriptomic resources in all organisms, including non-model species, enables the identification of genes involved in secondary metabolism pathways^8,20^. Recent NGS platforms have revolutionized sequencing methods to identify uncharacterized genes/proteins involved in complex pathways^7^. For instance, using the de novo method, Verma, et al.^1^ investigated the transcripts of leaf, flower and root tissues in *C. roseus*, resulting in the identification of 59,220 unique transcripts with an average length of 1284 bp. Almost 65% of the transcripts showed homology to the deposited sequences in the databases, while 35% of the unigenes were specific to the bisindole alkaloids synthesis activity in the plant leaves and roots organs, whereas enzymes associated with vindoline and vinblastine synthesis were solely found in the plant aerial tissues. Furthermore, Verma, et al.^1^ identified 11,620 simple sequence repeats (SSRs) markers and 1820 transcription factor (TFs) in *C. roseus*.

Kellner, et al.^7^ generated a partial genome assembly for *C. roseus* aiming to understand the monoterpenoid indole alkaloids (MIAs) biosynthesis pathway^7^. In the past 10 years,, not only the MIA pathway leading to the formation of strictosidine, but several downstream pathway branches, and previously undescribed TFs and transporters have been characterized^21,22^.

Jasmonic acid (JA) and salicylic acid (SA) among other elicitors have been shown to alter the expression of TIAs pathway genes^11,23,24^. It has already been reported that most alkaloid regulators in *C. roseus* are induced by JA. Nonetheless, they exert cell-specific effects, since some TFs exclusively regulate the specific part of the MIA pathway^21,22^.

It has been documented that SA induces the expression of TIAs pathway genes and the synthesis of vindoline, vinblastine and vincristine alkaloids^11,23,25,26^. Spraying SA on *C. roseus* leaves resulted in a significant increase in the expression of TIAs pathway genes, which in turn caused the accumulation of catharanthine, vinblastine and vindoline alkaloids^11,27^. Also, spraying SA on leaves in *C. roseus* plants facing water^28^ and salinity^25^ stresses, reduced the effects of stress and increased the total amount of alkaloids, more specifically a significant increase in vincristine, and vinblastine production rate. Nevertheless, despite the beneficial effects of the application of SA, higher concentrations (> 1 mM) of SA resulted in decreasing the amount of alkaloids, suggesting that the induction role of SA seems to depend on the concentration of SA^29^. Therefore, it can be anticipated that the expression pattern of associated TIAs pathway genes following SA treatments by RNA-Seq, will further facilitate the unravelling and identifying genes involved in the biosynthesis of complex molecules such as vincristine and vinblastine.

## Results

### Quantitative assessment, Sequencing and de novo assembly

In order to generate a comprehensive transcriptome of C. roseus leaves in response to 0.1 mM SA treatment 24-h post-treatment, cDNA libraries were constructed in three biological replicates and sequenced. In total, 191,022,812 bases were generated by RNA-Seq (Table 1). Mean unread nucleotides (N) were reported to be zero in all samples. The average length of the genes in all the libraries was 1277 bp. Protein coding genes were assessed using the BUSCO pipeline. It was shown that the majority of the eudicot’s core genes had been successfully recovered. Specifically, by identifying 2326 total BUSCO groups, 46.6% were complete single-copy, 45.1% were complete duplicated, 2% were fragmented, and 6.3% were missing.

**Table 1:** Trinity assembly stats report of assembled contigs.

Attribute	CD- Hit	Trinity
Total trinity genes	47,640	64,531
Total trinity transcripts	74,301	120,739
GC (%)	39.98	40.11
Contig N50	2201	2273
Average contig length	1479	1582
Total assembled bases	109,874,530	191,022,812

### Annotation and taxonomy of unigenes

**Figure 1:**
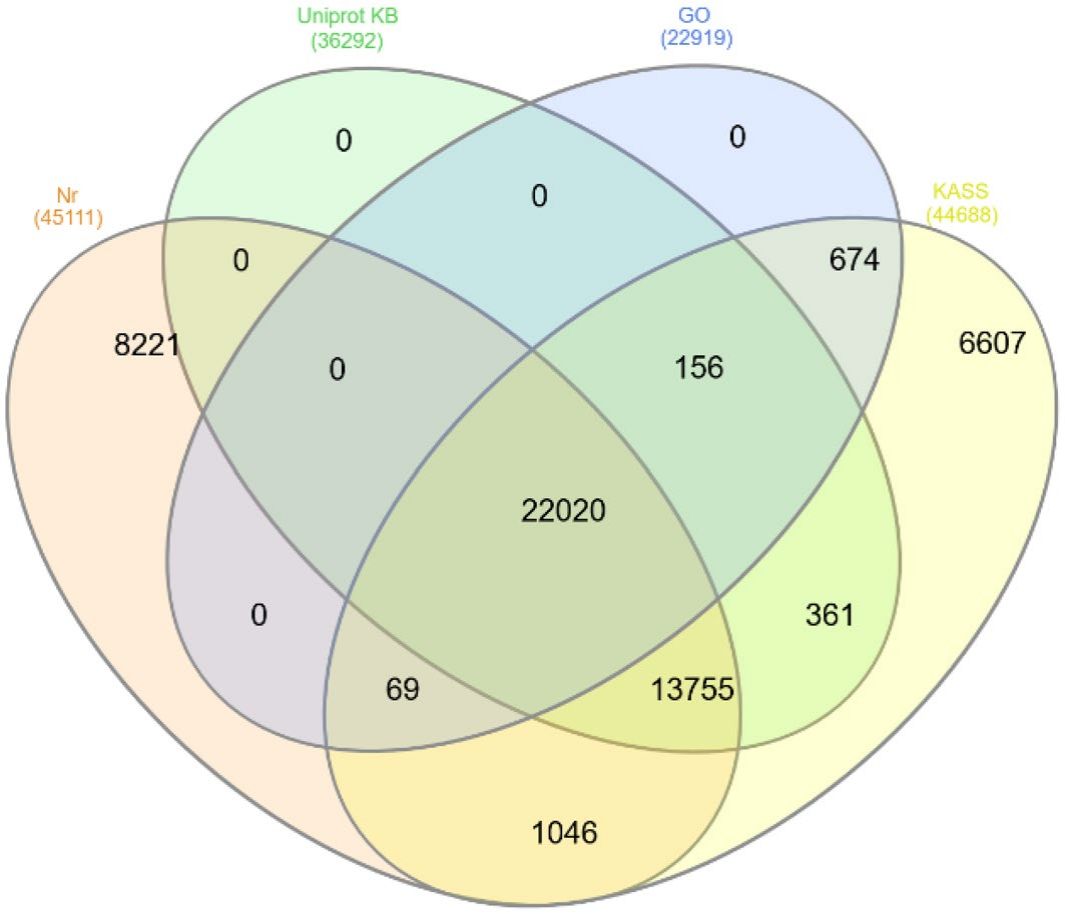
Venn diagram showing the number of differentially expressed transcripts homologous to known annotated genes from KASS, GO, Uniprot KB and NCBI non-redundant (NR) protein databases.

**Figure 2:**
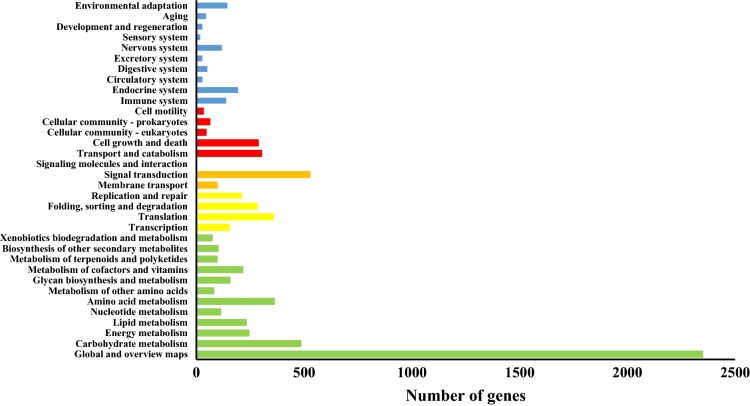
The result of annotation in GhostKOALA (green: Metabolism, yellow: Genetic Information Processing, orange: Environmental Information Processing, red: Cellular Processes and blue: Organismal Systems).

The unigenes’ annotation was done using BLAST against KAAS, Uniprot, and NCBI non-redundant (NR) protein databases. Out of 74,301 transcripts identified, 44,688 (60%), 36,292 (49%), 45,111 (61%), and 22,919 (31%) from all the libraries were annotated against KAAS, Uniprot, NCBI NR protein, and GO databases, respectively (Fig. 1). Also, the Blast results showed 63% and 64% similarity with *Arabidopsis thaliana* in BLASTP and BLASTX, respectively. From 56,939 inputs (Quarry), 47,398 (83%) unitranscripts were allocated to Eudicots based on GhostKOALA analysis^30^. According to GhostKOALA server, genetic information processing had the highest number in the functional classification of Protein families. Sections such as secondary compounds, amino acid metabolism, and terpenoid metabolism were also identified (Fig. 2).

### Gene ontology (GO) classification and Functional KEGG analysis

To determine the functional classification of unigenes, WEGO was used^31^. Results of this type of analysis led to the identification of 55 functional categories. The unigenes were divided into three categories, including cellular components, molecular function, and biological processes. In the biological process category, metabolic processes, cellular processes and biological regulation were highly represented groups. Moreover, binding and catalytic activity constituted the largest proportion of molecular function and cellular component categories, respectively. Annotation of unigenes in the KEGG database was done using KAAS, and *Arabidopsis* as the reference genome. Metabolic pathways, biosynthesis of secondary metabolites, and biosynthesis of amino acids such as tryptophan metabolism, tyrosine metabolism, and phenylalanine metabolism pathways were identified (Supplementary 2).

### Identification and classification of differentially expressed unigenes

**Figure 3:**
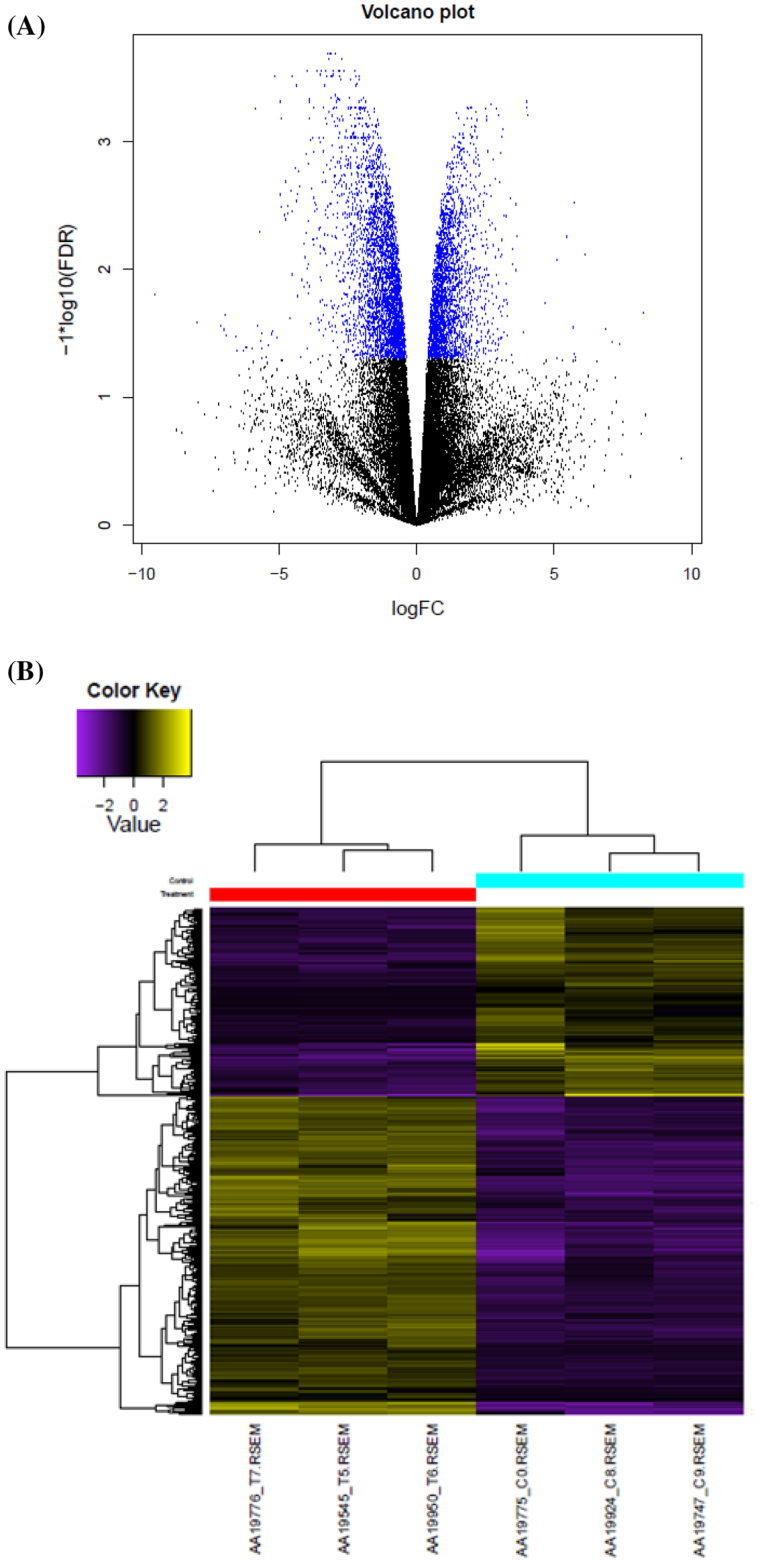
(**A**): A volcano plot illustrating the Log2-fold change between the treatments versus control plants. Blue dots represent significantly DEGs between treatment (24-h post 0.1 mM salicylic acid) and control (distilled water treatment) plants, (**B**)**:** Heat Map plot of differential expression genes between the treatment and controls.

Differential expression analysis was performed using EdgeR package with FDR < 0.05 and log_2_ fold-change|≥ 1|^32^. In total, 8348 DEGs between the control and SA-treated libraries were identified in which 3892 DEGs were up-regulated and 4456 DEGs were down-regulated (Fig. 3). At the 95% significance threshold, unigenes with different expression levels were identified (Supplementary 3).

### GO and Annotation of DEGs

Following identifying genes with different expression patterns between treatment and control samples, gene ontology was performed by goseq package^33^. The results of this analysis led to the identification of 81, 5 and 52 terms for biological processes, cellular components and molecular functions, respectively (Supplementary4). Among genes associated with biological processes, the GO terms related to biological process, metabolic process and biological regulation had a significantly higher level of expression.

The genes associated with metabolic pathways, biosynthesis of secondary metabolites, biosynthesis of amino acids, specifically tryptophan metabolism, tyrosine metabolism, and phenylalanine metabolism were differentially expressed. There were also differentially expressed unigenes related to the biosynthesis of terpenoid, monoterpenoid biosynthesis, diterpenoid biosynthesis, phenylpropanoid biosynthesis, flavonoid biosynthesis, flavone and flavonol biosynthesis, indole alkaloid biosynthesis, isoquinoline alkaloid biosynthesis, and tropane, piperidine and pyridine alkaloid biosynthesis (Supplementary 5).

### Genes associated with terpenoid compounds

**Figure 4:**
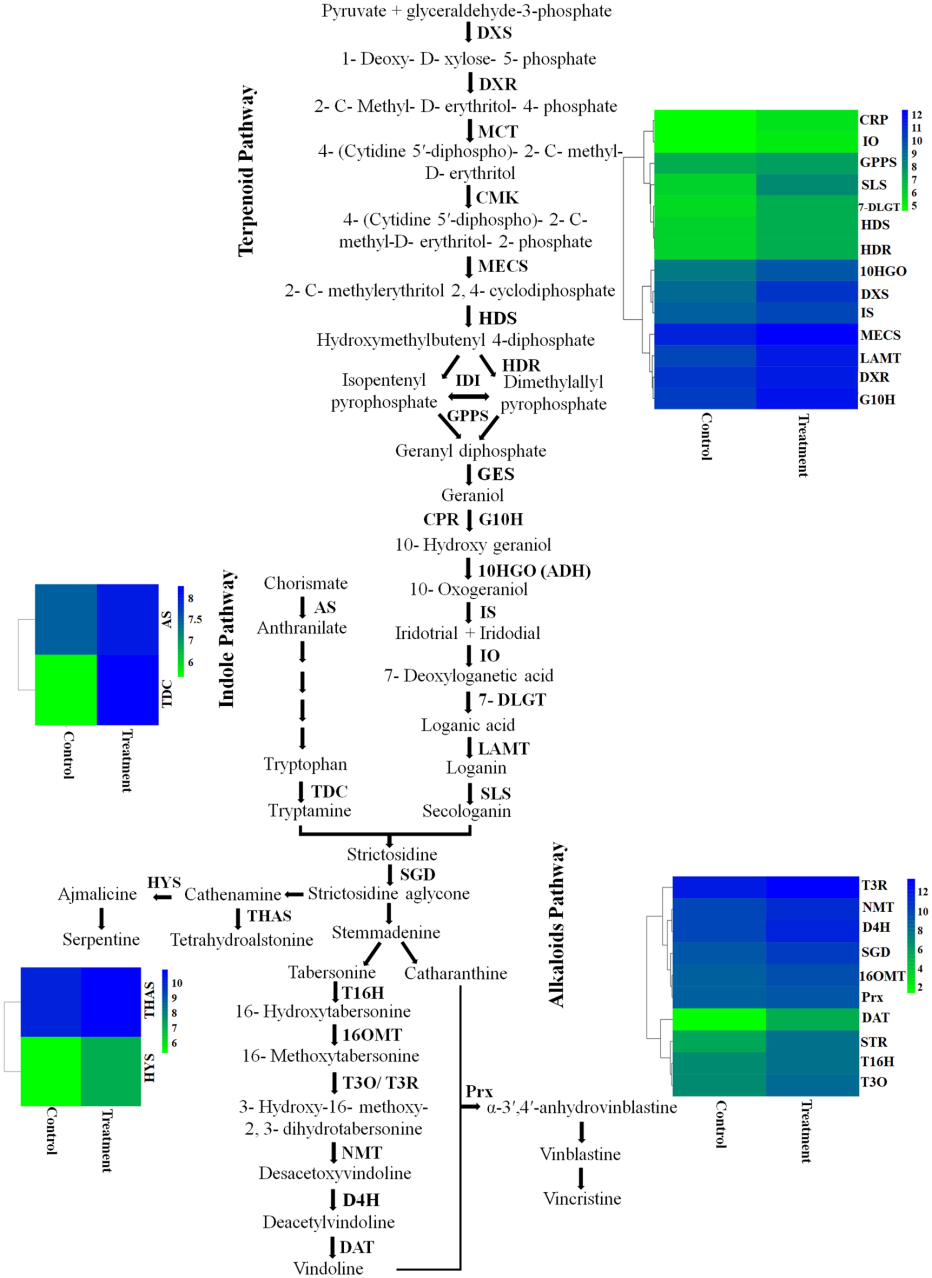
Expression patterns of *C. roseus* genes involved in the terpenoid indole alkaloids biosynthetic pathways.

In the pathway related to terpenoid compounds, 19 DEGs between control and treatment samples were identified, including *DXS*, *DXR*, *ISPE*, *ISPH*, *ISPF* and *GGPS*. These enzymes are at the beginning of the terpenoid pathway and are the most important enzymes in the biosynthesis pathways derived from the Methyl Erythritol Phosphate (MEP) pathway. Information and names of genes with different expressions in the biosynthetic pathway of the aromatic amino acids tryptophan, phenylalanine and tyrosine can be seen in supplementary 5. The results of this analysis showed differences in the expression of anthranilate synthase and tryptophan synthase genes. Also, the chorismate mutase gene was differentially expressed. Differences in the expression of genes related to alkaloid compounds were also noticed. The production of indole terpenoid alkaloids is specific only to *C. roseus* and is not synthesized in other plants such as *Arabidopsis*, which has been used as a template in the analysis. For this reason, the protein sequences of TIAs pathway enzymes were obtained from the UniProtKB/Swiss-Prot. The *C. roseus* database was prepared for the TIAs pathway and the sequence of transcripts was compared and analyzed. As a result of this analysis, all genes in the TIAs pathway were identified (Fig. 4).

### Validation of DEGs by qRT-PCR

**Figure 5:**
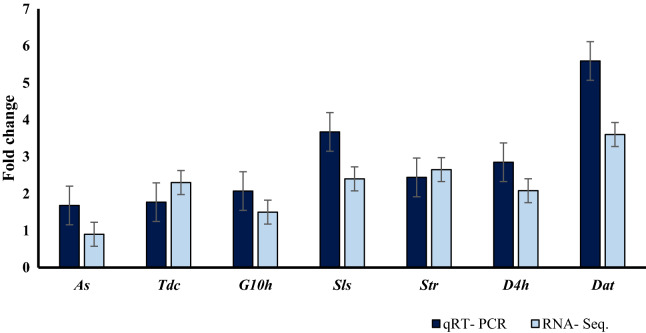
Comparison of qRT-PCR^27^ and RNA-Seq of *Tdc*, *G10h*, *Sls*, *Str*, *D4h* and *Dat* genes in 24-h treatment.

To confirm and validate the accuracy and reliability of the transcriptome analysis data, seven candidates unigenes associated with the TIAs pathway were chosen for qRT-PCR analysis. The results of qRT-PCR analysis for selected hub genes were previously published^27^. However, to avoid repeated sampling errors, relative fold changes were used from our previous study. Consistent with RNA-Seq analysis, fold change expression of DEGs of the qRT-PCR analysis for selected key genes of the TIAs pathway showed an increase in expression following SA treatment plants in comparison to the control sample (Fig. 5).

### Vinblastine and Vincristine content

**Figure 6:**
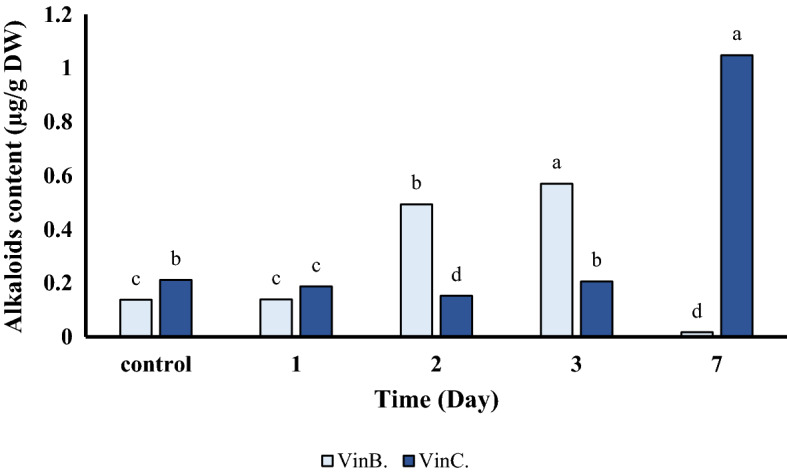
Effect of spraying of salicylic acid on vinblastine and vincristine content of *C. roseus*.

Vinblastine and vincristine are effective agents for treating various types of cancers, including leukemias, lymphomas, and other solid tumors^34^. To evaluate the effect of SA treatment on the production rate of these alkaloids, the amount of vinblastine and vincristine was measured at 1st, 2nd, 3rd and 7th days post-treatment by HPLC analysis (Fig. 6). It was found that SA treatment was effective in increasing the amount of vincristine, the final product of the TIAs pathway. As it can be seen from Fig. 6, SA treatment led to a significant increase in vincristine production. Although the amount of vinblastine significantly increased on day 2nd and 3rd post SA treatment, on day 7th the amount of vinblastine dropped sharply.

### Identification of TFs and SSR markers

Overall, we found 1895 TFs of which 710 TFs had a differential expression, belonging to different families. bHLH, C3H, , C2H2, MYB, MYB-relatedAP2/ ERF, NAC, bZIP, and WRKY were the most common TFs (Supplementary 6).

The *C. roseus* transcript sequences were searched for microsatellite loci using the MIcroSAtellite identification tool (MISA)^35^. Overall, 77,192 SSRs were identified. Among these SSRs, the di-nucleotides and tri-nucleotide SSRs were the most abundant SSR markers, accounting for 39% and 34% of the total SSR markers, respectively (Supplementary 7). There was a low number of hexa- (5%) and panta-nucleotide (4%) SSRs.

## Discussion

Omics data for medicinal plants is limited in comparison to crop plants. Transcript analysis by NGS techniques has made it possible to identify genes associated with pathways involved in the production of valuable alkaloids^8^. *C. roseus* is one of the most important medicinal plants that is considered as a model plant for secondary metabolome studies^36^. Apart from environmental and culture conditions, elicitation is also considered to be an important factor in promoting alkaloid production in medicinal plants. For instance, the use of fungi/bacteria as elicitors for improving alkaloid production in many medicinal plants has been well documented^11,23,37,38^. Identification and annotation of alkaloid biosynthesis in *C. roseus* are of immense importance. Gene-to-metabolite networks in *C. roseus* cells have shown that genes play a major role in metabolites accumulation^16^. Salicylic acid has been reported to affect the accumulation of *C. roseus* metabolites such as vindoline, vincristine, and vinblastine^11,25^. Consequently, exogenous application of SA can increase the rate of alkaloid production in many plant species. For instance, medicinal plants subjected to SA treatments display a wide range of complex molecular responses, including changes in gene expression, modulation of transcriptional regulation, and signal transduction related to secondary metabolites pathways^39–43^. In this study, treatment of *C. roseus* leaves with SA resulted in altering the expression of major genes involved in indole terpenoid alkaloids biosynthesis. The amount of vincristine production was also significantly increased following SA treatment, whereas vinblastine accumulation decreased dramatically on day 7th. This could be due to the vinblastine has been converted to vincristine at high capacity on day 7th^2,6,38,44^. Currently some partial RNA-Seq data of *C. roseus* are available where one can search for key genes associated with metabolic pathways leading to the production of important alkaloids^1,8,45,46^. Despite many efforts to identify key regulators of TIAs biosynthetic pathway, there are ambiguities regarding intermediate compounds of TIAs biosynthetic pathway^1,8^. We performed de novo transcriptome analysis using the Illumina HiSeq 2500 platform to investigate and identify functional genes in response to SA treatment in *C. roseus*. The results showed that 83% of the transcripts assigned to the dicots. More than 60% of unigenes were annotated in databases (Fig. 1) with the highest similarity of 64% to *Arabidopsis*. Similarly, transcriptome analysis of *C. roseus* revealed 38,380 annotated unigenes with the highest similarity to *Arabidopsis* transcriptome^1^. The results of comparing de novo transcript on medicinal plants database against *C. roseus* showed 94% similarity. Carbohydrates, flavonoids, saponin and alkaloids are the main constituents of *C. roseus* secondary metabolites^2,38^. Consequently, the highest percentage of annotated genes belonged to biological functions. Similarly, Verma, et al.^1^ and Pan, et al.^8^ identified the same pathways following treatment of *C. roseus* plants with methyl jasmonate. *C. roseus* terpenoid indole alkaloids are derived from the MEP and shikimate pathways^2,44^. The product of the terpenoid and indole pathways enters the synthesis pathway of terpenoid indole alkaloids with the activity of the STR enzyme. Results of this study showed that the expression of genes involved in alkaloids biosynthesis in *C. roseus* changed following salicylic acid treatment. qRT-PCR analysis showed that SA treatment increased the expression of genes associated with the synthesis of terpenoid indole alkaloids (Fig. 5). Consequently, up-regulation of these genes led to vincristine and to a lesser extent vinblastine production (Fig. 6).

It was found that many of up-regulated genes in *C. roseus* in response to SA treatment, were key receptors/signaling genes or were associated with SA-mediated responses. The results of this study have confirmed the results of previous studies by indicating the positive effect of SA treatment on genes associated with TIAs pathway and its end product, vincristine. As far as the literature is concern, until now the comparison between salicylic acid and MeJA’s influence on the expression of genes involved in alkaloids biosynthesis in *C. roseus* has not been reported. However, separate pieces of research have been conducted to assess the effect of MeJA and JA on the expression of genes associated with TIAs pathway. For instance, Wei (2010)^47^ showed that MeJA treatments induced the expression patterns of TIAs pathway genes in *Catharanthus roseus* seedlings. Even though MeJA and JA seem to share similar effects on the overall expression of TIAs genes, according to Rischer et al.(2006), using *C. roseus* cell suspension cultures showed that JA induced the expression of some TIAs biosynthetic genes, such as *G10H*, *SGD*, and *TDC*, but the expression of genes encoding *MAT* and *DAT* did not change. Taken together and in comparison to our results, MeJA, JA and SA treatments seem to target similar TIAs genes expression. However, further analysis is needed to unveil the effects of all three organic compounds in a single study at the same time.

TFs play an important role by modulating the expression level of their target genes. NGS methods with the aim of bioinformatics analysis have made it possible to identify both TFs and target proteins in different species^6,48^. In the present study, 1895 TFs were identified that 710 transcription factors had differential expression. bHLH, C3H, C2H2, MYB, MYB-related, AP2/ERF, NAC, bZIP and WRKY families had the highest number of TF members in *C. roseus*^6^. Interestingly, Verma, et al.^1^ identified bHLH, MYB, AP2/ ERF and WRKY TFs in a *C. roseus* transcriptome study. Also, the study of the effect of methyl jasmonate and ethylene on the synthesis of TIAs led to the identification of WRKY and ABC TFs^8^. It is well documented that the synthesis of terpenoid indole alkaloids is modulated by TFs related to the structural genes of the TIAs pathway^6,49,50^. For example, two TFs, ORCA2 and ORCA3, as members of the AP2/ ERF transcription factor family, are crucial regulators TIAs Pathway^6,51^. The overexpression of ORCA2 significantly increased the expression of many genes in the TIAs pathway resulting in a higher accumulation of catharanthine, ajmalicine, serpentine, tabersonine^52^ and vindoline^53^. In addition to the role of TFs belonging to the AP2/ ERF family, it has been shown that CrMYB2 overexpression in the hairy roots of *C. roseus* alters the expression of ORCAs and also increases the amount of catharanthine and tabersonine^54^. In fact, the bHLH transcription factor regulates the expression of structural genes, whereas ORCA3 does not regulate structural genes and may play redundant roles^22^. WRKY1 transcription factor binds to the Wbox element in the promoter of the *Tdc* gene, regulating the expression level of *As*, *Dxs*, *Sls*, *Sgp* and *Tdc* genes, consequently, and modulating the production of serpentine and decreased catharanthine. Therefore, WRKY is a good candidate for regulating metabolites such as serpentine and ajmalicine production^6,14^.

SSR markers are co-dominant species-specific markers which are used in breeding programs to study genetic diversity, and kinship relationships germplasm analysis^55,56^. Out of 77,192 SSR markers identified by MISA, three and two nucleotide repeats had the highest number of SSRs with 34% and 39%, respectively. The presented results were consistent with the results of Verma, et al.^1^ where the highest percentage of SSRs belonged to two and three nucleotide repeats.

## Conclusion

In addition to the importance of the *C. roseus* as the only source of vinblastine sulfate and vincristine sulfate drugs, it is also considered as a model plant in studying secondary metabolites production. This is the first study dealing with the expression analysis of TIAs pathway genes following SA treatment. The expression of all genes in the TIAs pathway producing anti-cancer alkaloids vinblastine and vincristine increased after SA treatment. These results were consistent with the measurements of vincristine alkaloid (the final product of the TIAs pathway) over time and showed that SA spray had a positive effect on both transcription and production of alkaloids. The result of this study can be used as a way to increase the anti-cancer alkaloids in *C. roseus*.

## Materials and methods

**Figure 7:**
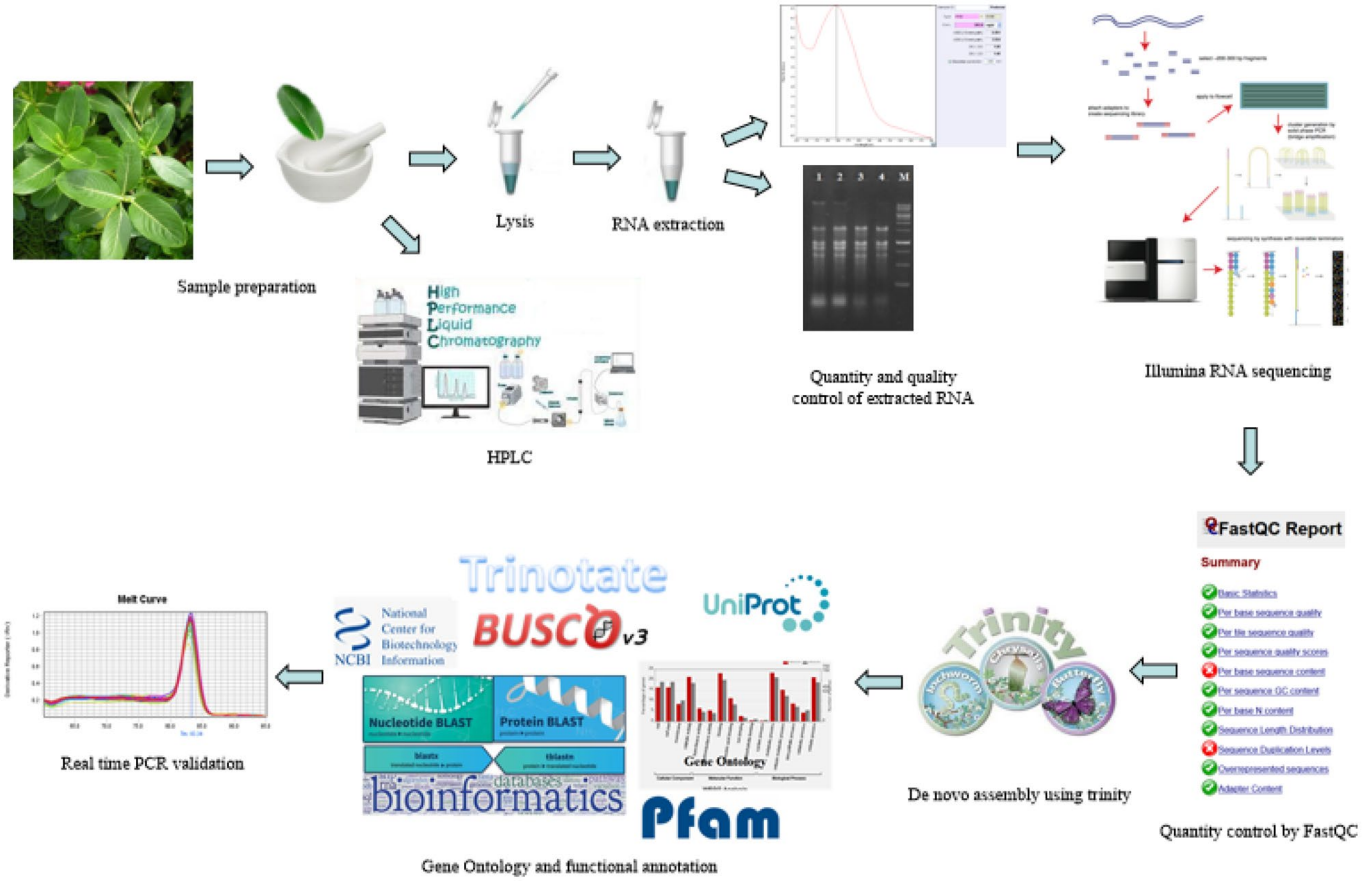
Schematic illustration of different steps taken for analysis.

The pipeline of study is shown in Fig. 7.

### Plant material, growth conditions, and treatment

**Figure 8:**
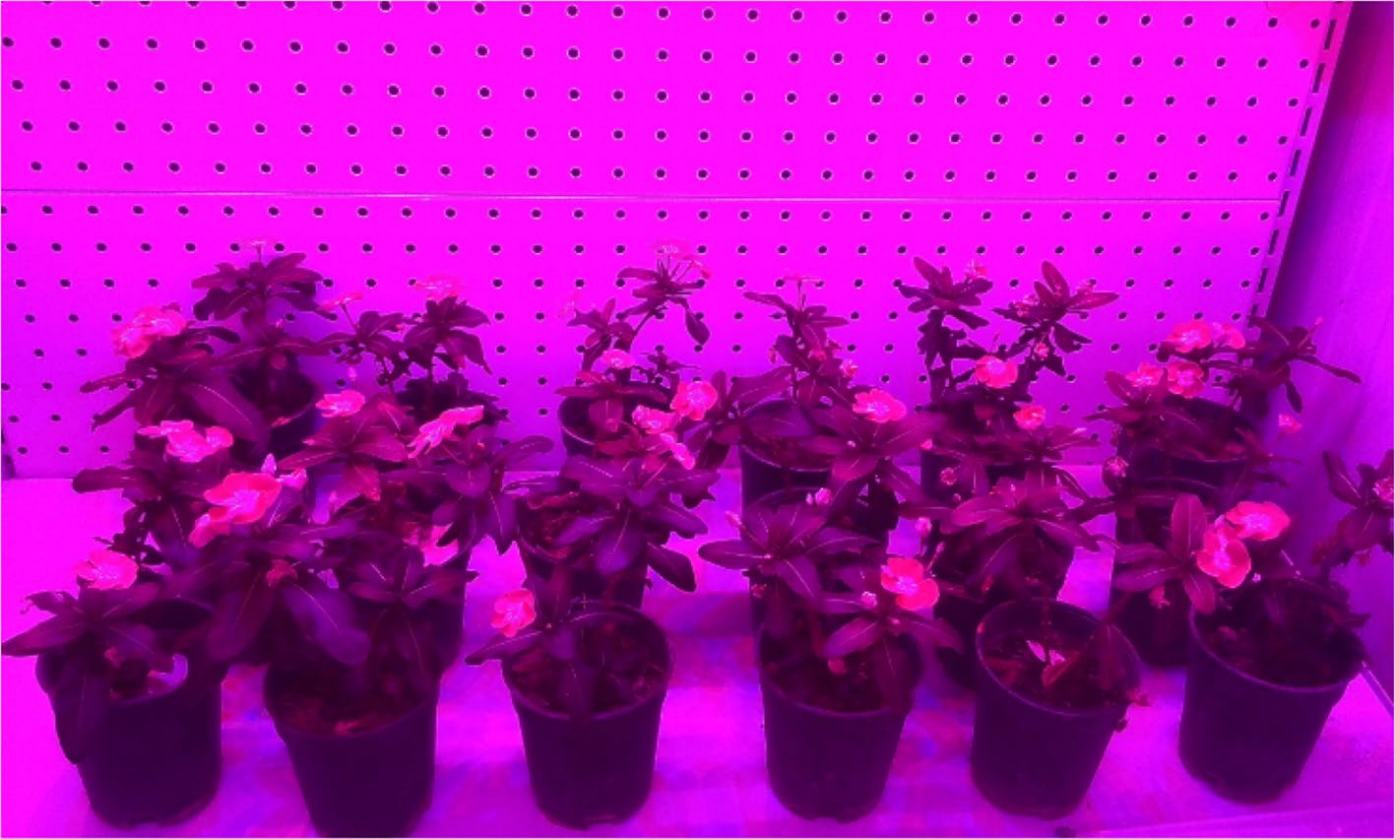
Seedlings of C. *roseus*.

Periwinkle (*Catharanthus roseus* (L.) G. Don ‘Pacifica XP Burgundy Halo Vinca’ seeds were purchased from Pan American Seed Company (USA, SKU: 506,521). The *C. roseus* seeds were planted in pots (10 cm diameter × 10 cm height) and kept in a growth chamber (25 °C, 16 h photoperiod, and light intensity of 70% µmol m^−2^ s^−1^). At the beginning of flowering stage (Fig. 8) as recommended^2,57^, SA has sprayed on the leaves at concentrations of 0.01 and 0.1 mM^23^. Leaf samples were collected from the third and fourth leaves according to Roepke, et al.^58^ at 12, 18, 24, and 48 h post-treatment (3 replicates each). The collection of plant material, complies with relevant institutional, national, and international guidelines and legislation. Plant materials were then immediately frozen in the liquid nitrogen and stored at − 80 °C for RNA extraction. Based on the results of qRT-PCR analysis from our previous study^27^ (Supplementary 1), 24-h post SA treatment (SA: 0.1 mM salicylic acid) and control plants (C: distilled water treatment) were selected for RNA-Seq.

### RNA preparation, cDNA library construction and sequencing

Total RNA was extracted from leaves using the Monarch® Total RNA Miniprep Kit (NEB Company, UK. Cat# T2010) according to the manufacturer’s instructions. The extracted RNA samples were treated with DNase I enzyme to remove possible DNA contaminations. The quantity and quality of RNA samples were analyzed by using the NanoDrop 2000c Spectrophotometer (Thermo Scientific NanoDrop 2000, USA) and 1% agarose gel electrophoresis, respectively. Next, high-quality RNA samples (three biological replicates), were sent to the sequencing company (Beijing Genomes Institute, China). Furthermore, quality controls and the RNA integrity number (RIN) were assessed for all RNA samples. cDNA preparation and sequencing were performed with the use of standard Illumina kits according to the manufacturer’s instructions (Beijing Genomes Institute, China). Finally, paired-end sequencing was performed by Illumina HiSeq 2500 platform with a read length of 150 nucleotide.

### Quality control, processing and de novo assembly

Sequencing data quality assessment was performed by using FastQC 0.11.20^59^. Trimmomatic 0.36^60^ was employed to remove adapters, nucleotides and low quality readings as well as to check the quality of the nucleotides reads. De novo assembly was conducted using Trinity 2.11.0^61^ by default parameters, k-mer length of 32 and minimum transcripts length of 300 bp. Trinity transcripts were clustered using CD-HIT by identity cutoff of 95%^62^. Transcriptome completeness was assessed using the bioinformatics tool BUSCO v3 (Benchmarking Universal Single-Copy Orthologs) to obtain the percentage of single-copy orthologues represented in three datasets edicots_odb10^63^. After integrating the readings, to identify the candidate Coding Sequences (CDS), all sequences were translated into protein sequences by TransDecoder tool (http://transdecoder.github.io). To find homology for identified transcripts and translated peptides, BLASTX and BLASTP were performed, respectively.

### Functional annotation of C. roseus assembled unigenes

Using a Trinotate annotation pipeline (http://trinotate.github.io/), functional annotation of the *C. roseus* transcriptome was done. Next, the assembled unigenes of *C. roseus* were blasted against NR proteins (http://www.ncbi.nlm.nih.gov/) and UniProtKB/Swiss-Prot (https://www.uniprot.org/) databases. Then, HMMER software was used to predict protein domains against the Pfam database. Also, signal peptide and transmembrane domains were identified using the SignalP5.0 server. Biological pathway information (Available in the Kyoto Encyclopedia of Genes and Genomes (KEEG) database) using GhostKOALA (KEGG Orthology And Links Annotation)^30^ (https://www.kegg.jp/blastkoala/) and KEGG Automatic Annotation Server (KAAS) (https://www.genome.jp/kegg/kaas/) servers, gene ontology terms and functional classifications (biological, cellular, and molecular) related to identified homologous genes were extracted from the WEGO (Web Gene Ontology Annotation Plot) Web tool (http://wego.genomics.org.cn/).

### Differential gene expression analysis

Initially, all high-quality readings were aligned with Bowtie2 software against the assembled transcript. Then, the frequency of integrated copies was estimated using RSEM^64^. EdgeR package was used to normalize and obtain the differences in gene expression. Next, using the goseq package^33^ in R software 4.1.0^65^, ontological analysis and classification of terms into cellular, molecular and biological categories for genes with different expressions was performed. Finally, the KASS database was used to annotate unigenes with different expressions.

### Differential gene expression related to terpenoid indole alkaloids (TIAs) pathway

Protein sequences related to the *C. roseus* TIAs pathway (available in the UniProtKB/Swiss-Prot database) were obtained. Differentially expressed transcripts were blasted against the *C. roseus* protein database to re-evaluate the expression changes of the genes in the target pathway. Finally, the diagram of the genes involved in the TIAs pathway was plotted.

### qRT- PCR validation

For qRT- PCR validation, Total RNA extraction and cDNA synthetize were carried out. The full details of the qRT-PCR analysis and the results were published in Soltani et al.^27^.

### Determination of alkaloid contents

The samples were prepared according to Singh, et al.^66^ with slight modifications. Briefly, leaf samples were dried in the shade and pulverized leaf tissues were three times suspended in 80% methanol solution. After drying, 10 ml of water was added to the samples and the pH was adjusted to 3.5. The aqueous phase was removed by washing three times with chloroform, the pH was raised to 8.5 by adding ammonia and after three washes with chloroform, the chloroform phase was removed. Methanol was added to the dried sample and stored in the refrigerator for further analysis.

### HPLC analysis

High-performance liquid chromatography was performed using Eurospher II 100–5 C18 column with precolumn (Column 250 * 4.6 mm) and UV detector (model K-2600) with an HPLC device (KNAUER WellChrom model) at 254 ɳm. The mobile phase consisted of methanol: diammonium dihydrogen phosphate (5 mM) with a composition of 71:29 (pH = 7.3) and a flow rate of 1 mL/min^67^.

### Statistical analysis

One-way analysis of variance (ANOVA) was done by using Agricolae package 1.3–5^68^ in R software 4.1.0^65^ to statistically compare changes of vinblastine and vincristine alkaloids content at the different sampling points. The Least Significant Difference (LSD) test was employed to compare treatment means at a confidence of 95%.

### Identification of TFs and SSRs

For the identification of *C. roseus* TFs families, the TF database Plant TFDB 5.0 was used^69^. The MISA web (https://webblast.ipk-gatersleben.de/misa/)^35^ was used to identify the *C. roseus* microsatellites.

## Supplementary Information

Supplementary Information.

## Data Availability

All the Illumina sequencing reads have been deposited in the National Genomics Data Centre with the accession code (BioProject ID: PRJNA830709). https://dataview.ncbi.nlm.nih.gov/object/PRJNA830709?reviewer=2nim3dscqae7pc7vrvkvj8gens.
